# Synergistic metabolism of salivary MUC5B in oral commensal bacteria during early biofilm formation

**DOI:** 10.1128/spectrum.02704-23

**Published:** 2023-10-19

**Authors:** Carolina Robertsson, Gunnel Svensäter, Julia R. Davies, Anders Bay Nord, Daniel Malmodin, Claes Wickström

**Affiliations:** 1 Department of Oral Biology, Faculty of Odontology and Biofilms Research Center for Biointerfaces, Malmö University, Malmö, Sweden; 2 Swedish NMR Centre, Gothenburg University, Gothenburg, Sweden; The Ohio State University College of Dentistry, Columbus, Ohio, USA

**Keywords:** bacterial metabolism, dental biofilm, metabolomics, NMR, biofilm physiology, MUC5B, oral microbiology, streptococci, actinomyces, saliva

## Abstract

Bacterial metabolism in oral biofilms is comprised of complex networks of nutritional chains and biochemical regulations. These processes involve both intraspecies and interspecies networks as well as interactions with components from host saliva, gingival crevicular fluid, and dietary intake. In a previous paper, a large salivary glycoprotein, mucin MUC5B, was suggested to promote a dental health-related phenotype in the oral type strain of *Streptococcus gordonii* DL1, by regulating bacterial adhesion and protein expression. In this study, nuclear magnetic resonance-based metabolomics was used to examine the effects on the metabolic output of monospecies compared to dual species early biofilms of two clinical strains of oral commensal bacteria, *S. gordonii* and *Actinomyces naeslundii*, in the presence of MUC5B. The presence of *S. gordonii* increased colonization of *A. naeslundii* on salivary MUC5B, and both commensals were able to utilize MUC5B as a sole nutrient source during early biofilm formation. The metabolomes suggested that the bacteria were able to release mucin carbohydrates from oligosaccharide side chains as well as amino acids from the protein core. Synergistic effects were also seen in the dual species biofilm metabolome compared to the monospecies, indicating that *A. naeslundii* and *S. gordonii* cooperated in the degradation of salivary MUC5B. A better understanding of bacterial interactions and salivary-mediated regulation of early dental biofilm activity is meaningful for understanding oral biofilm physiology and may contribute to the development of future prevention strategies for biofilm-induced oral disease.

**IMPORTANCE:**

The study of bacterial interactions and salivary-mediated regulation of early dental biofilm activity is of interest for understanding oral microbial adaptation to environmental cues and biofilm maturation. Findings in oral commensals can prove useful from the perspectives of both oral and systemic health of the host, as well as the understanding of general microbial biofilm physiology. The knowledge may provide a basis for the development of prognostic biomarkers, or development of new treatment strategies, related to oral health and disease and possibly also to other biofilm-induced conditions. The study is also an important step toward developing the methodology for similar studies in other species and/or growth conditions.

## INTRODUCTION

In prokaryotic as well as eukaryotic metabolism, enzymatic and non-enzymatic reactions convert metabolites for biosynthesis, energy production, or functional alteration in response to external signals (1), such as fluctuations in pH or receptor-ligand interactions. Metabolic networks can be viewed as functional modules, where the central carbon metabolism is an example of a module that is highly conserved between organisms, while there are also large numbers of secondary, or specialized, networks that differ hugely between species (1, 2). Synergistic conversion of metabolites between the members of a biofilm also increases the diversity of the metabolic output (1, 3). Information about complex cellular activities and mechanisms for adaptation can therefore be deduced from studying transitioning metabolomes, and measuring biochemical phenotypes is one of the aims in metabolomics (1, 4).

Regulation of bacterial processes in oral biofilms is complex because it involves both intraspecies and interspecies networks as well as interactions with components from host saliva, gingival crevicular fluid, and dietary intake (3, 5). One salivary component that plays a major structural role in salivary films as well as the liquid phase (6, 7), and has been found to maintain oral health in various ways, is the gel-forming mucus glycoprotein, MUC5B (8, 9). Like other mucins at mucosal interfaces, MUC5B defends mucosal integrity as part of innate immunity through general physiochemical functions such as lubrication, diffusion limitation, and pellicle formation, as well as antimicrobial defense (10). MUC5B modulates bacterial activity by agglutination and modification of biofilm activity by provision of specific nutrients of glycan and protein origin for oral bacteria (8, 10 – 12). The mucin domains of MUC5B are highly glycosylated with O-linked oligosaccharide side chains consisting of fucose, galactose, N-acetylglucosamine, sialic acid, and N-acetylgalactosamine monosaccharides (13, 14). These domains are then flanked by lateral regions of exposed protein core rich in a variety of amino acids such as proline, serine, and threonine but also valine, arginine, and glutamic acid with some N-linked oligosaccharides (13, UniProt accession number Q9HC84). These components are available for utilization as nutrients for bacteria with mechanisms for monosaccharide and disaccharide or amino acid release and uptake (15).

The lifestyle in biofilms enables various interactions within and between its members such as signal and response, co-adhesion, and co-habitation within a common extracellular matrix (16). Development of metabolic synergies drives the maturation of oral biofilms, and the collective capacity of microbial communities to extract energy from available nutrients far exceeds the metabolic capabilities of any species in isolation (16). *Actinomyces* and *Streptococcus* are two of the main genera identified in oral biofilms (17), and they have been found to co-aggregate through specific cell surface receptor binding (18). Despite the many similarities in the metabolic machinery of these mainly saccharolytic genera, *Actinomyces* and *Streptococcus* also exhibit some key differences, e.g., in their arrays of enzymes for carbohydrate uptake and metabolism (19, 20). The spatial cell-cell proximity that follows co-aggregation between these bacteria facilitates synergistic effects where these organisms can take advantage of each other’s sets of enzymes to perform more elaborate metabolic and other cellular interactions, e.g., enhance biofilm formation and growth, and thereby increase the competitiveness of this intergeneric pair in mixed biofilms (21).

The two oral species *Actinomyces naeslundii* and *Streptococcus gordonii*, which the current study focuses on, both represent early colonizing oral commensals (22) with similar, but in part significantly different, enzymatic profiles. Both strains are part of a four-species consortium previously shown to collectively be able to break down salivary MUC5B (9). In one of our previous studies, salivary MUC5B was suggested to promote a dental health-related phenotype of the oral *Streptococcus S. gordonii* by regulating bacterial protein expression, largely by down-regulating proteins involved in carbohydrate uptake and acid production, while also reducing the attachment of the more caries-related *Streptococcus mutans* compared to *S. gordonii* (23). The current paper compares the metabolomic profiles of early monospecies and dual species biofilms of *A. naeslundii* and *S. gordonii* grown with salivary MUC5B to study how these two strains may complement each other in the degradation of such a complex substrate within a dual species biofilm. Collaboration between early colonizers to degrade complex substrates such as salivary MUC5B to release nutrients for microbial utilization may hugely impact biofilm physiology over time, and is thereby highly associated with oral health factors related to preventing transition to dysbiosis in maturing biofilms which may otherwise cause biofilm-induced disease (9, 16). A better understanding of host mechanisms that preserve oral health through the salivary-mediated regulation of dental biofilm activity is meaningful for understanding oral biofilm physiology. Effects on metabolomic profiles in oral commensals in response to salivary MUC5B may be associated with regulatory events with roles in sustaining eubiosis in oral biofilms.

## MATERIALS AND METHODS

### Enrichment of human salivary MUC5B for conditioning films

Non-stimulated whole saliva was collected on ice from nine healthy individuals and pooled. To isolate MUC5B, isopycnic density gradient centrifugation was performed as described previously (24). In brief, the pooled saliva was diluted 1:2 in 0.2 M NaCl and solubilized overnight by gentle stirring. The pooled solution was then centrifuged for 30 min at 4,400 × *g*, 4°C (Beckman Coulter Avanti J-E centrifuge, JA 20 rotor), for the removal of debris. The starting density of the supernatant was then set to 1.45 g/mL with CsCl, followed by ultracentrifugation for 96 h at 36.000 rpm, 15°C (Beckman Coulter Optima LE-80K Ultracentrifuge, 50.2 Ti rotor). Twenty-four 1.7 mL fractions were then collected from the top of the tubes and pooled separately. Antibodies for the MUC5B polypeptide backbone central domain (6F10-E4, Novus Biological) were utilized for enzyme-linked immunosorbent assays to detect enriched fractions. These fractions were pooled separately to produce a MUC5B-enriched solution. The solution was then dialyzed against 10 mM phosphate buffer with 0.07 mM NaCl (PBS) (Spectra/PorTM Dialysis Membrane Biotech CE tubing, MWCO: 100 kDa) and stored at −80°C. The protein concentration was then measured by freeze drying and weighing after dialysis against water for removal of salts, and determined to be 0.3 mg/mL.

### Bacterial isolates

The bacteria used in this study were clinical isolates employed in previous studies (9, 15), isolated from dental plaque of a healthy individual. After selection of the strains from samples of dental biofilms based on morphology and routine streptococcal and actinomyces identification protocols, both strains were sequenced for identification with 16S rRNA Sanger sequencing after polymerase chain reaction (PCR). DNA was extracted with chemical, enzymatic, and mechanical cell lysis steps. The 16S rRNA genes were amplified using universal bacterial 16S rRNA primers (forward primer 8F, AGAGTTTGATCCTGGCTCAG; reverse primer 1492, GGTTACCTTGTTACGACTT, Sigma-Aldrich). The PCR products were sent to Eurofins Genomics for purification and Sanger sequencing. The 16S rRNA sequences were then aligned *in silico* using ARB software (25), identified by BLAST search alignment (NCBI) and published in GenBank (accession numbers OQ625896 for *Actinomyces naeslundii* CW and OQ625895 for *Streptococcus gordonii* CW).

### Glycosidase profiles

Glycosidase activity was tested using kits with 4-methylumbelliferyl-tagged enzyme substrates according to the manufacturer’s instruction (Sigma-Aldrich) for nine glycosidases with relevance for the degradation of salivary MUC5B oligosaccharide sidechains (α-L-fucosidase, β-D-fucosidase, α-galactosidase, β-galactosidase, α-mannosidase, β-mannosidase, β-N-acetylgalactosaminidase , β-N-acetylglucosaminidase and sialidase). Briefly, *A. naeslundii* CW and *S. gordonii* CW were grown on blood agar overnight in 5% CO_2_ at 37°C. Cultures were then dispersed in PBS, diluted 1:1 in working solutions of the enzyme substrates in 50 mM 2-{[1,3-dihydroxy-2-(hydroxymethyl)propan-2-yl]amino}ethane-1-sulfonic acid (TES buffer) pH 7.5 in duplicates and incubated in a 96-well plate for 1 h at 37°C on a slow shaker. After incubation, the end-point fluorescence was measured in each well (excitation λ 355 nm, emission λ 560 nm, CLARIOstar Plate reader, BMG Labtech).

### Biofilm cell cultures

*S. gordonii* CW and *A. naeslundii* CW cultures from blood agar were inoculated in 25% Todd-Hewitt Yeast Extract (¼ THYE, Becton Dickinson) and grown overnight in 5% CO_2_ at 37°C. The next day, the cultures were washed by centrifugation (3,000 rpm, 5°C, 10 min, 50 mL tubes, Beckman GS-6R centrifuge) and resuspended in sterile PBS pH 7.5 to an optical density measured at 600 nm (OD_600nm_) of 0.5 (corresponding to similar cell counts between the two species, 1.5 × 10^8^ CFU/mL for *S. gordonii* CW and 1 × 10^8^ CFU/mL for *A. naeslundii* CW). Twelve well plates and Ibidi VI 0.1 μ-slides (Ibidi GmbH, Munich, Germany) were pre-coated with MUC5B-conditioning solution with 0.1% wt/vol CaCl_2_ (final concentration), diluted in PBS 1:5 and incubated at room temperature overnight. Biofilm formation was initiated by inoculation of the PBS at an OD_600nm_ of 0.5 bacterial suspensions (separately for monospecies biofilms or 1:1 vol/vol of each culture for dual species, to give a final volume of 2 mL in 12-well plates and 100 µL in Ibidi channels per biofilm) to the mucin-coated wells and incubated in 5% CO_2_ at 37°C for 2 h to allow the cells to adhere. After the adhesion phase, buffer supernatant and non-adherent cells were removed by gentle rinsing with PBS and replaced with 25% MUC5B in PBS and incubated for 2 h as described above.

### Biofilm viability, surface coverage, species distribution, and biomass

Viability, surface coverage, and species distribution in three biologically independent replicates of monospecies and dual species biofilms from independent inoculates of *S. gordonii* CW or *A. naeslundii* CW cultured according to protocol was assessed after incubation with mucin in the Ibidi VI 0.1 μ-slides. To examine viability and surface coverage, biofilms were stained using the BacLight LIVE/DEAD viability kit (Invitrogen, Carlsbad, CA) and imaged at 60× magnification in a Nikon Eclipse TE2000 inverted confocal scanning laser microscope (CSLM) (Nikon Corp., Tokyo, Japan). An argon laser (488 nm laser excitation) with long-pass 515/30 (green fluorescence signal) and 605/75 (red fluorescence signal) filters was used for illumination. To assess species distribution in biofilms, cell cultures were pre-treated with CellTrace Cell Proliferation Kits before inoculation to Ibidi slides. Cultures in PBS (OD_600nm_ of 0.5) were stained with 1 µM CellTrace Far Red Dye (*A. naeslundii* CW) or 5 µM CellTrace CFSE Green Dye (*S. gordonii* CW) according to the manufacturer’s protocol with minor adaptations as described here. After addition of CellTrace dye working solutions, cultures were incubated at 37°C for 1 h protected from light, washed (Eppendorf centrifuge 5415 D, 12.000 rpm at room temperature (RT)), resuspended in sterile PBS 1:1 vol/vol and then inoculated to produce biofilms as described under the Biofilm cell cultures section. CellTrace-labeled biofilms were then imaged at 60× magnification using a Nikon Eclipse TE2000 spinning disc confocal microscope with a CFI Plan Apokromat 60× oil lens, numerical aperture 1.40 (Nikon), Prime 95B Scientific CMOS camera (Photometrics), and SPECTRA X light engine (Lumencor Inc.) for illumination.

Each biofilm triplicate was imaged at 10 different randomly selected positions. Image analysis was performed using the BioImage_L software package (26). The viability assay was performed by calculating the percentage green pixels (LIVE/DEAD), surface coverage from the total percentage of pixels with signal (LIVE/DEAD), and species distribution by calculating the percentage green and red pixels (CellTrace) in each image.

To monitor the biomass between replicate biofilms, a crystal violet biofilm quantification assay was performed for all replicates at the end of each experiment. After collection of supernatants and sampling for culture to exclude contamination and confirm survival of both species, all wells were fixed by incubation with 99% ethanol for 30 min. The wells were then air dried for 10 min, rinsed three times gently with sterile PBS, and stained with 0.2% crystal violet solution in PBS for 5 min. Excess stain was then removed by rinsing with PBS. The stain retained in the biofilms was then dissolved in 33% acetic acid, and the absorbance was read at 570 nm.

### Nuclear magnetic resonance (NMR) sample preparation, acquisition setup, data processing, and metabolite annotation

After incubation, the supernatants were collected, centrifuged for 5 min at 5,000 rpm, 4°C (Heraeus Fresco 17 centrifuge, Thermo Scientific), and moved to fresh tubes to remove suspended cells. The supernatants were then stored at −20°C until shipment to the Swedish NMR Centre in Gothenburg for NMR analysis, where frozen samples were thawed on ice. A total volume of 300 µL of each sample was transferred manually to a deep well plate (Porvair catalog number 219030) prefilled with 300 µL buffer {75 mM sodium phosphate, pH 7.4, 0.08% wt/vol TSP-d4 [3-(Trimethylsilyl)propionic-2,2,3,3-d 4 acid sodium salt], 0.1% wt/vol sodium azide, 20% vol/vol deuterium oxide} in each well. After sealing with a silicone lid, the plate was shaken at 500 rpm, 12°C for 5 min on a Thermomixer Comfort (Eppendorf). A SamplePro Tube L (Bruker Biospin) liquid handler was used to transfer 575 µL of each mixed sample to 5 mm SampleJet NMR tubes (Bruker Biospin). NMR data were acquired from five biologically independent replicates on a Bruker Avance NEO 600 MHz spectrometer equipped with a 5 mm QCI cryoprobe and a cooled SampleJet sample changer. Sample racks were kept at 6°C during sample preparation as well as in the spectrometer sample changer up until measurement.

The standard pulse sequence “zgespe” was used to acquire 1D 1H data. The experiment encompassed a perfect echo sequence with excitation sculpting for water suppression. A total of 64 scans were collected into 64 k data points with a spectral width of 11,904 Hz, using an acquisition time of 2.692 s, a relaxation delay of 4 s, and eight dummy scans. The receiver gain was set to a fixed value of 101 and acquisition was done at 25°C. The acquired data were zero-filled twice and an exponential line broadening of 0.3 Hz added before Fourier transform and subsequent automated phasing and baseline correction. Spectra were referenced to the TSP-d4 signal at 0 ppm. Data acquisition and processing were performed in TopSpin 4.1.4 (Bruker BioSpin). Metabolite signal annotation was performed in ChenomX 9.0 (ChenomX Inc.).

The NMR data were imported into Matlab [MATLAB version: 9.13.0 (R2022b), Natick, Massachusetts: The MathWorks Inc.; 2022] using the function rbnmr (27). The TSP-d4 peak was aligned between all spectra using the function ico_shift (28) and set to 0 ppm. The data were then imported to R using the package R.matlab. Corresponding metadata was imported using the package readx1. The data were processed with the package speaq (29). Peaks were picked with the getWaveletPeaks function with baselineThresh = 0, SNR.Th = 10, and include_nearbyPeaks = TRUE. Peaks were grouped with the PeakGrouper function with min.samp.grp = 10 and grouping.window.width = 200. The SilhouetR function was run and groups with a value less than 0.5 were listed to be regrouped in a second run. The PeakFilling function was run filling in missing values with max.index.shift = 200. The output list was manually curated while it was annotated leaving 27 peaks for analysis of the MUC5B samples.

### Pathway enrichment analysis

Pathway enrichment analysis was performed using the Kyoto Encyclopedia of Genes and Genomes (KEGG) database (30) and MetaboAnalyst 5.0 (Montreal, QC, Canada) (4) against *Streptococcus pyogenes* M1 476 (serotype M1) reference metabolome (30), which was the closest related species to *S. gordonii* and *A. naeslundii* available in the database. In the analysis, the identified compounds were tested against annotated pathways in the reference metabolome to detect over-representations compared to what would be expected by chance based on the uploaded compound list.

### Statistical analyses

For the biofilm viability assay and surface coverage, statistical analysis of percentage green pixels (LIVE/DEAD stain) and percentage of total number of pixels with signal as calculated using the software package BioImage_L (26) from the replicate images was performed using one-way analysis of variance (ANOVA) and Tukey’s *post hoc* test with the significance level set to *P* < 0.05 (Fig. 3). For the assessment of species distribution in the dual species biofilms (CellTrace dye), the percentage coverage of the green (*S. gordonii* CW cells) and red (*A. naeslundii* CW) pixels was reported as a mean % ± one standard deviation (Fig. 4).

Metabolites were considered to be present in a group if they were found in ≥2 of the five replicates. Nonparametric comparison of means between metabolite abundances in replicate groups of all three biofilm types was performed using the related-samples Friedman’s two-way analysis of variance by ranks with Bonferroni correction for multiple tests (SPSS), and comparison between metabolites only present in two conditions was performed using the related-samples Wilcoxon signed rank test. Paired replicate analyses were selected to compensate for technical batch effects between the different runs. Metabolite covariation analysis of intensity measured by NMR was done using an overview principal component analysis (PCA) made in Simca (Version 17.0.0.224543) (31) with confidence intervals of each group calculated in Matlab. The pathway enrichment analysis was based on Fisher’s exact test with false discovery rate analysis to correct for multiple tests.

## RESULTS

The aim of this study was to investigate the effects on the metabolic output in monospecies compared to dual species early biofilms of two clinically isolated oral commensal bacteria, *S. gordonii* CW and *A. naeslundii* CW, in the presence of the complex salivary glycoprotein MUC5B.

### Bacterial adherence of monospecies biofilms to MUC5B-coated surfaces

**Fig 1:**
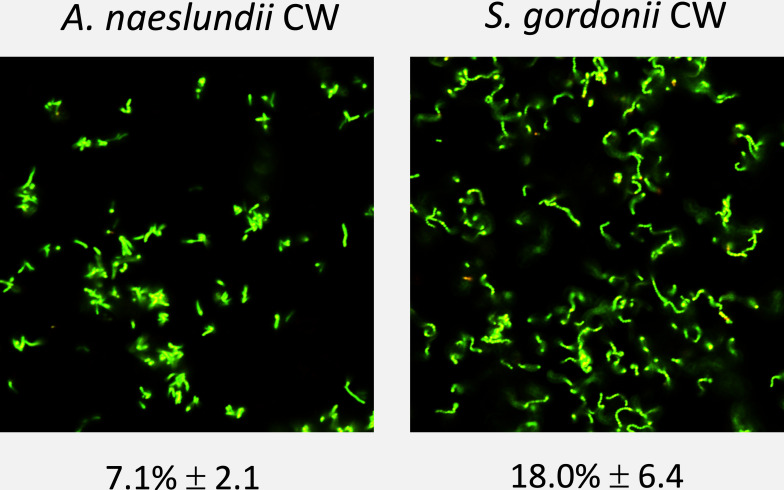
Mean percentage biofilm surface coverage ± one standard deviation of monospecies *A. naeslundii* CW and *S. gordonii* CW early biofilms on salivary MUC5B, imaged using confocal scanning laser microscopy (BacLight LIVE/DEAD stain).

Both *A. naeslundii* and *S. gordonii* are considered early oral colonizers, but differed significantly in their ability to adhere to salivary MUC5B (Fig. 1). The mean percentage surface coverage ± one standard deviation after the adhesion phase (Fig. 3) was 7.1% ± 2.1 in the *A. naeslundii* CW monospecies early biofilms (biofilm viability 96.3% ± 1.8), which was significantly lower than the surface coverage of *S. gordonii* CW (18.0% ± 6.4, viability 93.7% ± 4.4).

### Bacterial adherence of dual species biofilms to MUC5B-coated surfaces

**Fig 2:**
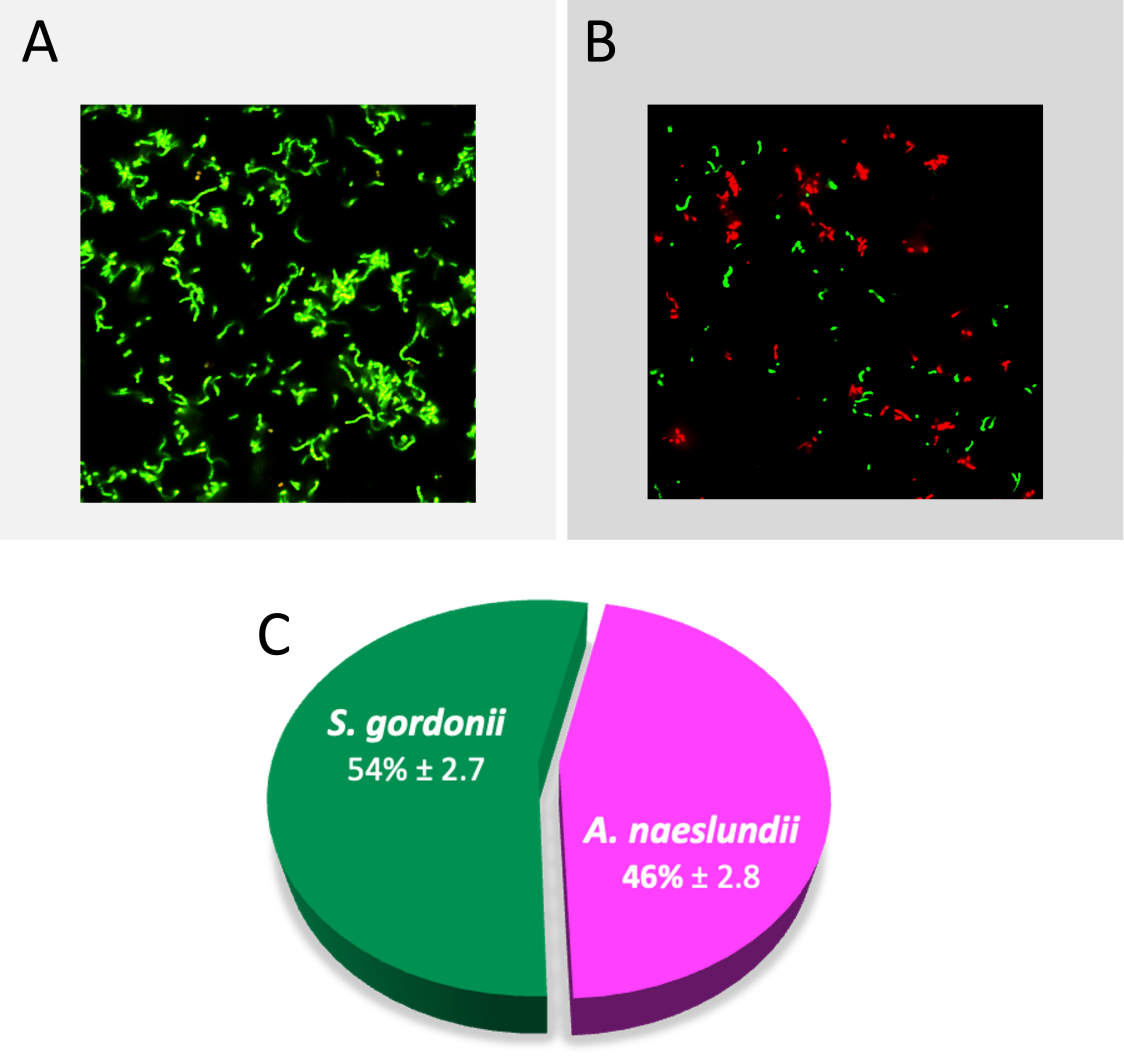
Dual species biofilm viability, surface coverage, and species distribution in early biofilms on salivary MUC5B, imaged using confocal scanning laser microscopy. (**A**) BacLight LIVE/DEAD stain (green, viable; red, dead), (**B**) CellTrace Far Red (red, *A. naeslundii* CW) and CFSE (green, *S. gordonii* CW) dye, (**C**) Pie chart of species distribution in dual species biofilm, mean percentage ± one standard deviation.

**Fig 3:**
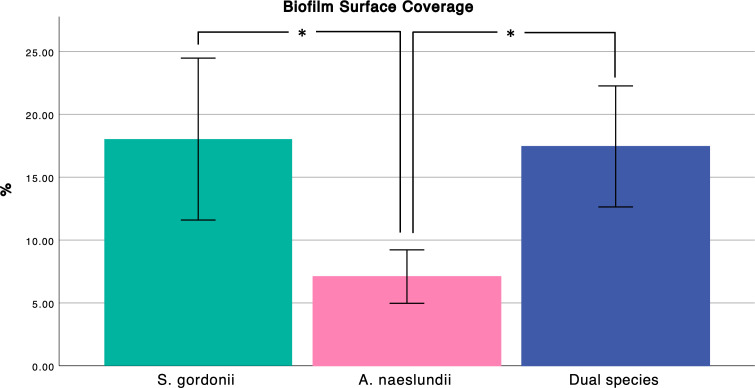
Biofilm surface coverage from image analysis of BacLight LIVE/DEAD stained biofilms imaged with CLSM. Significance tested with one-way ANOVA and Tukey’s *post hoc* test. Error bars show mean absorbance ± one standard deviation.

In the dual species early biofilms (Fig. 2A and B), the total surface coverage was similar to the *S. gordonii* monospecies biofilms, and significantly higher than the *A. naeslundii* biofilms (Fig. 2C). The surface coverage of these early biofilms was 17.4% ± 4.8 (Fig. 3, biofilm viability 97.4% ± 0.7). The proportion of the two species in the dual species biofilms was approximately equal (46.0% ± 2.8 for *A. naeslundii* CW and 54.0% ± 2.7 for *S. gordonii* CW), suggesting that *S. gordonii* promotes the colonization of *A. naeslundii* on salivary MUC5B.

### Biomass of attached cells

**Fig 4:**
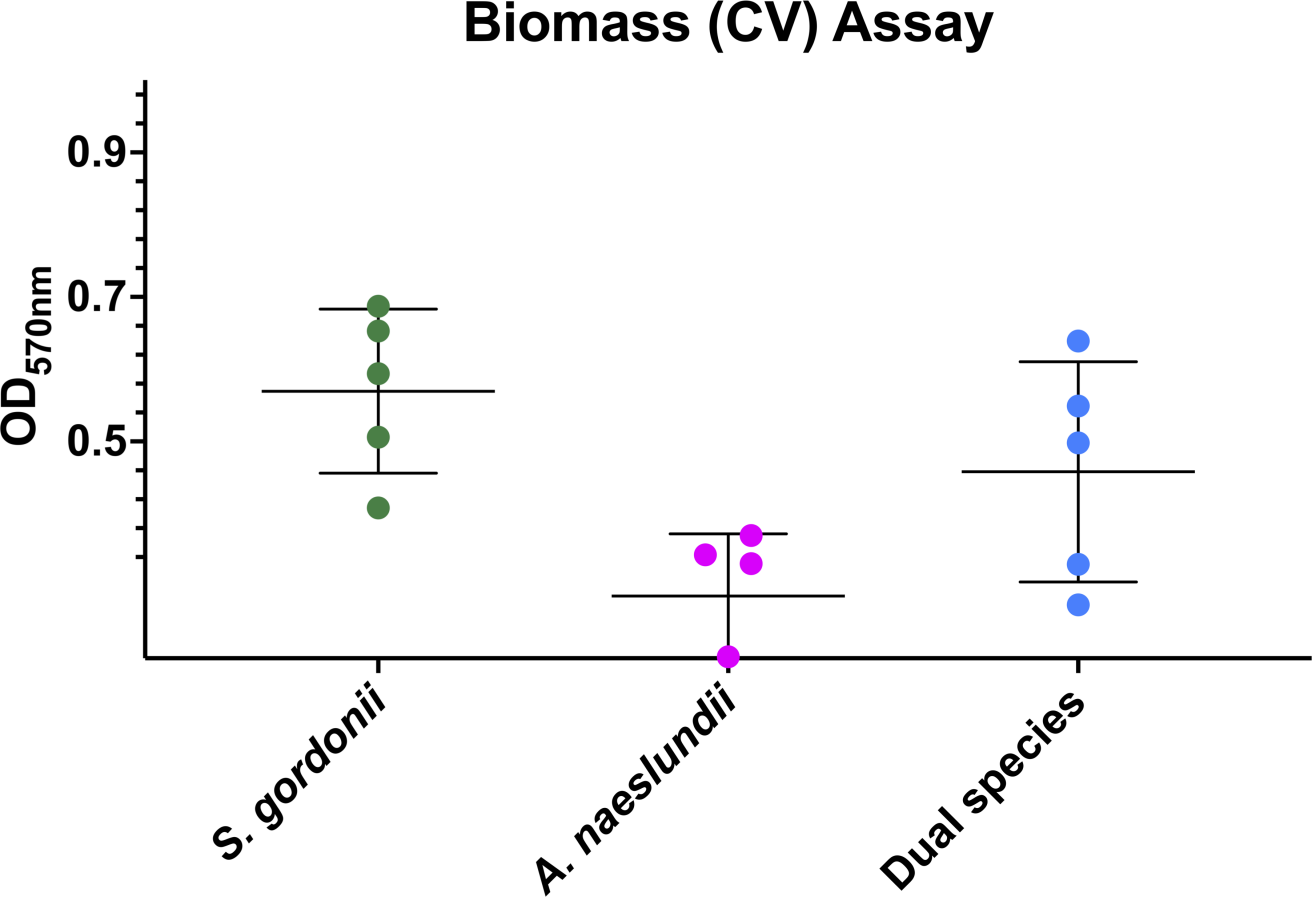
Absorbances read at 570nm from the crystal violet biomass quantification assay of all replicates at the end of each experiment. Error bars show mean absorbance± one standard deviation.

The crystal violet assay showed that the biomass of attached cells within replicate early biofilms was relatively consistent and that the differences in adherence (Fig. 2 and 3) were maintained until the end of the experiments (Fig. 4).

### Glycosidase profiles of *A. naeslundii* and *S. gordonii*

In this study, salivary MUC5B was the sole nutrient source during biofilm formation. Since the mucin domains of MUC5B carry numerous oligosaccharide sidechains that can be cleaved to generate monosaccharides and disaccharides for utilization as carbon sources for biofilm bacteria, it was of interest to investigate the glycosidase profiles of *A. naeslundii* CW and *S. gordonii* CW. A panel of nine glycosidases of relevance for degradation of MUC5B carbohydrate chains was selected (Table 1). *A. naeslundii* CW was positive for α-galactosidase, β-galactosidase, and sialidase, and *S. gordonii* CW was positive for α-L-fucosidase, β-N-acetylgalactosaminidase, and β-N-acetylglucosaminidase. The complementary glycosidase profiles that were displayed by the two commensals showed potential for synergistic degradation of MUC5B carbohydrate chains, which would make the complex salivary MUC5B available as a nutrient source during biofilm formation and growth. To investigate the fate of these mucin-derived carbohydrates in the bacterial metabolism, with special focus on dual species synergism, the metabolomes of monospecies and dual species early biofilm supernatants of *A. naeslundii* CW and *S. gordonii* CW formed with MUC5B as their sole nutrient source were analyzed.

**TABLE 1:** Glycosidase expression profiles of *A. naeslundii* CW and *S. gordonii* CW

	*Streptococcus gordonii* CW	*Actinomyces naeslundii* CW
	Mucin-degrading glycosidases	
α-L-fucosidase	+	−
β-D-fucosidase	−	−
α-Galactosidase	−	+
β-Galactosidase	−	+
α-Mannosidase	−	−
β-Mannosidase	−	−
β-N-acetylgalactosaminidase	+	−
β-N-acetylglucosaminidase	+	−
Sialidase	−	+

### Biofilm metabolites

From the NMR analysis of early biofilm supernatants, 14 individual metabolites were identified and 13 additional peaks were detected (peak raw data is available through the MetaboLights online repository, accession number MTBLS8370). The additional 13 peaks could not be annotated but were still clearly distinguished from artifact peaks in the NMR data set. In total, 21 peaks were detected in the *A. naeslundii* early biofilm supernatants, 18 in *S. gordonii,* and 18 in dual species. The majority of the identified metabolites (acetate, acetone, butyrate, ethanol, formate, lactate, methanol, propionate, pyruvate, and succinate) represent well-known metabolic end products of carbohydrate metabolism by oral bacteria. The increased accumulation of these metabolites compared to the controls verifies that MUC5B was utilized as a carbon source during early biofilm development. In addition, three amino acids (valine, glutamate, and arginine) and one amino acid degradation product (2-oxoisocaproate) were identified.

**Fig 5:**
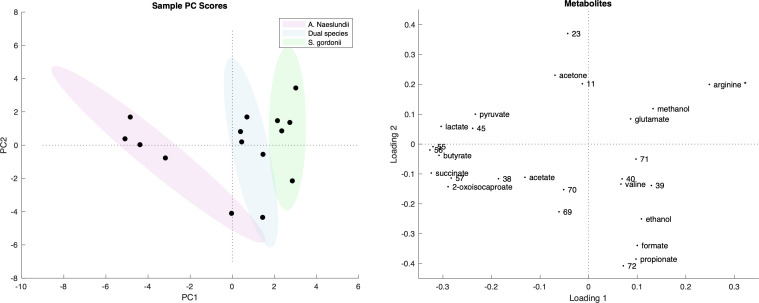
Principal component analysis comparing metabolomes from *A. naeslundii* and *S. gordonii* monospecies and dual species early biofilms grown in the presence of MUC5B. Score and loading plot of the first two components in a PCA with R2X(1) = 31.4% and R2X(2) = 17.0% variance explained. The sizes of the ellipses show the eigenvalues of the covariance matrix times the inverse of the χ^2^ cumulative distribution function of the scores at 95% confidence level. *, putative annotation.

In MUC5B, the monospecies and dual species early biofilms were clearly separated on the PCA (Fig. 5). Variation within groups was similar. The main metabolites that differentiated *A. naeslundii* from *S. gordonii* monospecies and dual species early biofilms were 2-oxoisocaproate, 57, succinate, 38, acetate, butyrate, 56, 55, lactate, 45, and pyruvate. *S. gordonii* biofilms were mostly differentiated by propionate, formate, ethanol, 39, 71, glutamate, methanol, and arginine. As expected, the overall dual species early biofilm metabolome was situated between those of the two monospecies on the PCA.

### Comparison of monospecies and dual species early biofilm shared metabolomes

**Fig 6:**
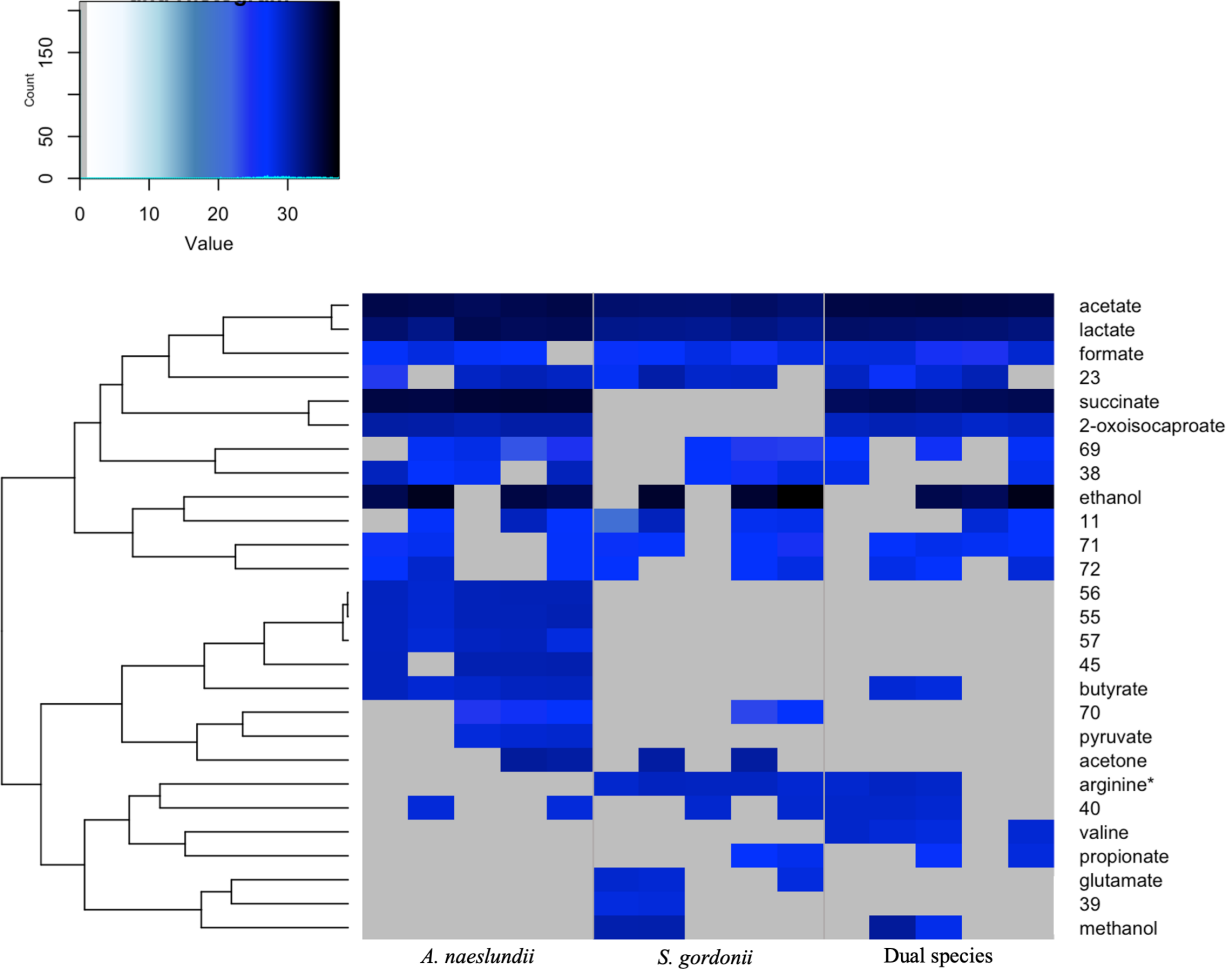
Comparison of metabolomes from *A. naeslundii* and *S. gordonii* monospecies and dual species early biofilms grown in the presence of MUC5B. Hierarchical clustering analysis of log2-normalized intensities for metabolites. Metabolites with signals that were detected in at least two of the five replicates within the groups are displayed.

Thirteen metabolites were common to both monospecies early biofilms, and 11 of these were present in both monospecies and dual species (Fig. 6). Of the 11 metabolites that were present in all three early biofilms (*A. naeslundii, S. gordonii,* and dual species)*,* acetate was significantly more abundant in the dual species early biofilms compared to *S. gordonii* monospecies (*P* = 0.005) even though the biomasses of these two biofilms were approximately the same (Fig. 3 and 4). Simultaneously, there was no significant difference in abundance of acetate between the two monospecies biofilms. Since the proportion of each species in the dual species biofilm was approximately the same, this suggests that the addition of *A. naeslundii* to the dual species biofilm gave rise to interactions that increased the amount of acetate present above the levels expected from the sum of the two monospecies.

**Fig 7:**
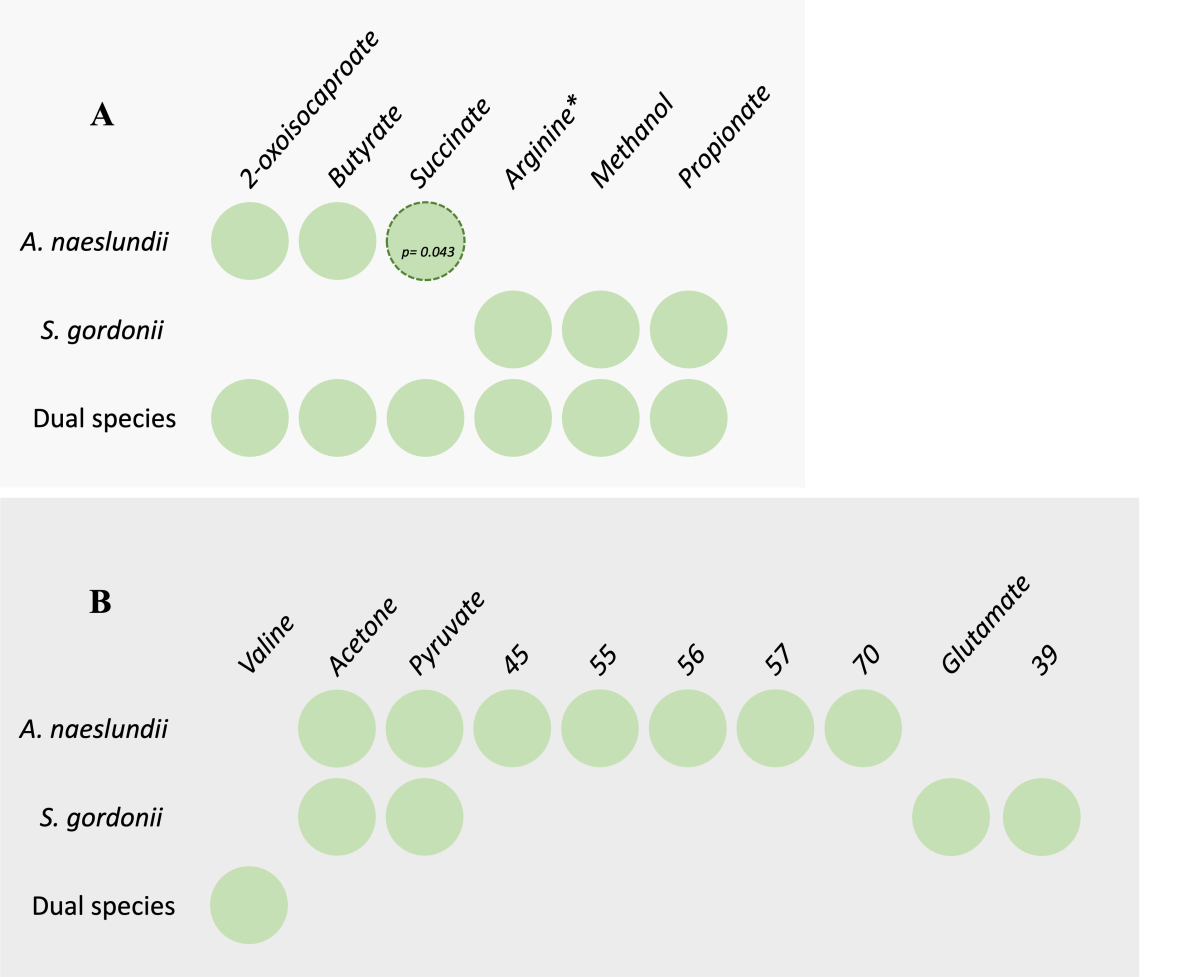
(**A**) Metabolites found in dual species produced by only one monospecies, (**B**) differences in metabolites present or missing in dual compared to monospecies metabolomes. Based on signals being detected in ≥2 of five replicates. Green, present; white, absent. Dotted outline, significantly more abundant, *P*-value is indicated.

Interestingly, lactate was significantly more abundant in *A. naeslundii* compared to *S. gordonii* monospecies early biofilms (*P* = 0.034), despite the lower biomass of the *A. naeslundii* biofilms. There was no significant effect on lactate abundance in the dual species early biofilms compared to the *A. naeslundii* biofilms despite an estimated increased amount of adherent *A. naeslundii* and larger total biomass in the dual species biofilms. The amount of lactate in the dual species was therefore reduced compared to what would be expected from the sum of monospecies.

Formate, ethanol, and metabolites 11, 23, 38, 40, 69, 71, 72 were also produced by all three groups but with no significant effects on abundance in the dual species early biofilms in relation to the biofilm biomass. This suggests a straightforward accumulative effect of the production from the individual species in the dual species biofilm. These metabolites represent a shared core metabolic output profile unaffected by dual species co-adhesion.

### Metabolites found in dual species biofilms but derived from only one species

In monospecies early biofilms, 2-oxoisocaproate, butyrate, and succinate were only produced by *A. naeslundii,* whereas arginine*, methanol, and propionate were only produced by *S. gordonii* (Fig. 6 and 7A). All of these metabolites, except succinate, were present in the dual species biofilms with no significant difference in abundance compared to the respective monospecies. However, succinate was significantly less abundant in the dual species biofilms compared to the monospecies *A. naeslundii* biofilms (*P* = 0.043), despite the increased proportion of *A. naeslundii*. This suggests that a synergistic effect caused a reduced accumulation of this metabolite in the dual species biofilms.

### Metabolites present or missing only in dual species

A number of metabolites were missing or only present in the dual species biofilms. Valine was found only in the dual species biofilms but was absent from both monospecies biofilms (Fig. 6 and 7B), and acetone and metabolite 70 were missing from the dual species biofilm while present in both monospecies biofilms (no significant difference in abundance). Pyruvate and metabolites 45, 55, 56, 57 were present only in the *A. naeslundii* monospecies biofilms, and glutamate and metabolite 39 were present only in *S. gordonii*, but absent from the dual species biofilms. Metabolites that are present or missing only in dual species biofilms compared to monospecies indicate synergistic effects of either metabolite co-regulation or secondary metabolic interplay between the two species.

### Metabolite covariation

A number of metabolites covaried to distinguish the monospecies and dual species early biofilms from each other in the PCA (Fig. 5). Five of the main metabolites that differentiated *A. naeslundii* monospecies early biofilms from *S. gordonii* and dual species (succinate, acetate, butyrate, lactate, and pyruvate) are involved in pyruvate metabolism. Three of the main metabolites that differentiated *S. gordonii* (propionate, formate, ethanol) are involved in pyruvate metabolism and two (glutamate, arginine*) are amino acids.

### Pathway enrichment analysis

**Fig 8:**
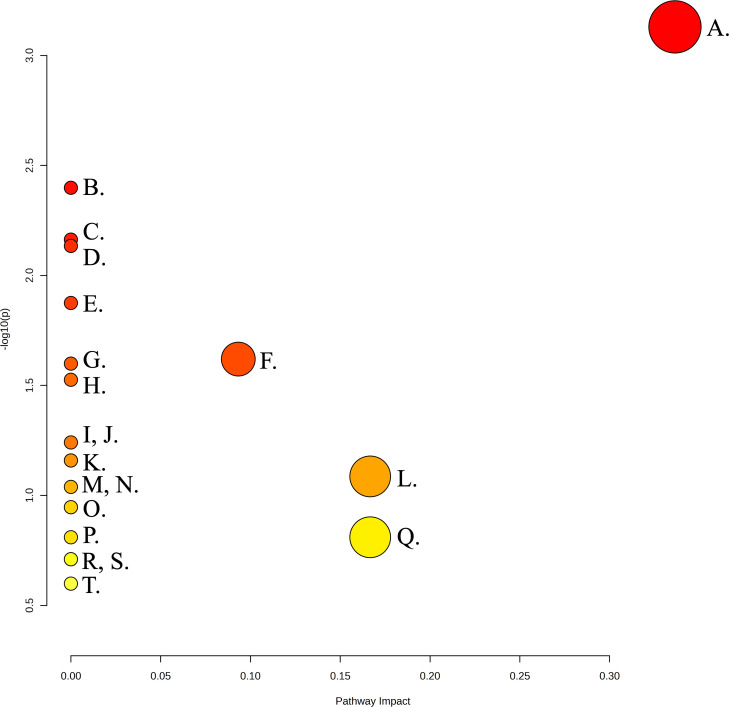
Graphical output of pathway enrichment analysis. Pathway impact, pathway impact score relative to the global reference metabolomic network. Created with MetaboAnalyst 5.0.

**Fig 9:**
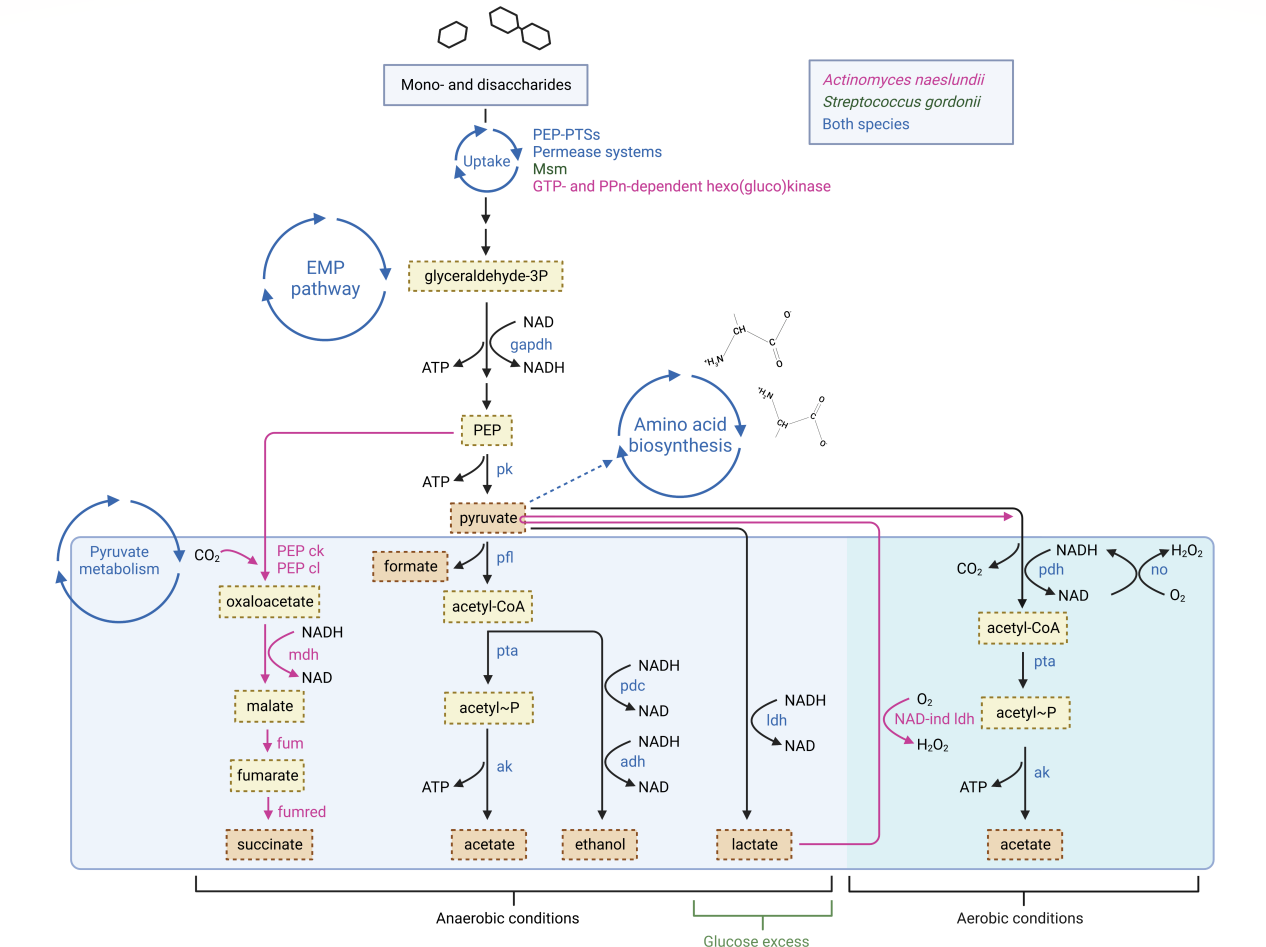
*A. naeslundii* and *S. gordonii* pyruvate metabolism. Metabolites identified in the NMR analysis are in darker shade boxes. Enzymes and processes are color coded; magenta, present in *A. naeslundii*; green, present in *S. gordonii*; blue, present in both species. PEP ck, phosphoenolpyruvate carboxykinase; PEP cl, PEP carboxylase; NAD/NADH, nicotinamide adenine dinucleotide; mdh, malate dehydrogenase; fum, fumarase; fumred, fumarate reductase; PEP-PTS, PEP-phosphotransferase system; Msm, multiple sugar metabolism transport systems; GTP, guanosine triphosphate; PPn, polyphosphate; EMP pathway, Embden-Meyerhof-Parnas glycolytic pathway; ATP, adenosine triphosphate; gapdh, glyceraldehyde 3-P dehydrogenase; pk, pyruvate kinase; pfl, pyruvate formate lyase; pta, phosphotransacetylase; ak, acetate kinase; pdc, pyruvate decarboxylase; adh, alcohol dehydrogenase; ldh, lactate dehydrogenase; pdh, pyruvate dehydrogenase; no, NADH oxidase; NAD-independent ldh, PEP-PTS, NAD-independent lactate dehydrogenase. Created with BioRender.com.

In the pathway enrichment analysis of all metabolites identified in early biofilms of *A. naeslundii, S. gordonii,* and dual species early biofilms formed in the presence of MUC5B, pyruvate metabolism was found to be significantly over-represented (Fig. 8; Table 2, *P* = 0.048). This was also the pathway with the highest pathway impact score (0.34). The metabolites matched to this pathway were acetate, ethanol, lactate, and pyruvate. Similarities and differences of *A. naeslundii* and *S. gordonii* pyruvate metabolism have been visualized in Fig. 9 [Adapted from previous studies (3, 19, 20, 32)]. The other metabolic pathways listed were also matched to compounds within the identified metabolome but showed no statistically significant over-representation.

**TABLE 2:** List of top 20 enriched pathways identified by pathway enrichment analysis, ranked by significance

Pathway	Total	Hits	−log10(p)	*P*-value (FDR)	Pathway impact	Metabolites
Pyruvate metabolism	20	4	3.130	0.048	0.34	Acetate, formate, lactate, pyruvate
Alanine, aspartate, and glutamate metabolism	15	3	2.398	0.119	0	Glutamate, pyruvate, succinate
Butanoate metabolism	18	3	2.163	0.119	0	Butyrate, succinate, pyruvate
Valine, leucine, and isoleucine biosynthesis	6	2	2.134	0.119	0	2-oxoisocaproate, valine
Arginine biosynthesis	8	2	1.874	0.173	0	Arginine*, glutamate
Glycolysis/Gluconeogenesis	28	4	1.620	0.234	0.093	Acetate, ethanol, lactate, pyruvate
Valine, leucine, and isoleucine degradation	11	2	1.600	0.234	0	2-oxoisocaproate, valine
Glyoxylate and dicarboxylate metabolism	12	2	0.030	0.242	0	Glutamate, pyruvate
Propanoate metabolism	17	2	1.241	0.410	0	Acetone, propionate
Carbapenem biosynthesis	3	1	1.159	0.410	0	Glutamate
Taurine and hypotaurine metabolism	3	1	1.159	0.410	0	Acetate
Aminoacyl-tRNA biosynthesis	45	3	1.086	0.444	0.167	Arginine, glutamate, valine
Tyrosine metabolism	4	1	1.039	0.457	0	Succinate
Histidine metabolism	5	1	0.946	0.490	0	Glutamate
D-Glutamine and D-glutamate metabolism	5	1	0.946	0.490	0	Glutamate
Citrate cycle (TCA cycle)	7	1	0.810	0.593	0	Pyruvate
Sulfur metabolism	7	1	0.810	0.593	0.167	Acetate
Glycine, serine, and threonine metabolism	9	1	0.710	0.667	0	Pyruvate
Arginine and proline metabolism	9	2	0.710	0.667	0	Arginine, glutamate
Nicotinate and nicotinamide metabolism	12	1	0.600	0.818	0	Succinate

^*a*^ Total, total number of compounds in the pathway; hits, number of metabolites from experimental data matched to the pathway; *P*-value (FDR), significance calculated from Fisher’s exact test and adjusted for multiple tests by false discovery rate (FDR); impact, pathway impact score relative to the global reference metabolomic network, calculated bioinformatically from pathway topology analysis based a number of importance measures; metabolites, name of metabolites that were matched to the pathway. *, putative metabolite identification. Created with MetaboAnalyst 5.0.

## DISCUSSION

In this study, analysis of NMR metabolomics data with interpretation of network integration was performed in order to investigate the effects of dual compared to monospecies composition of the two oral commensals *Actinomyces naeslundii* CW and *Streptococcus gordonii* CW on the early biofilm metabolomic profiles in the presence of salivary MUC5B. Metabolites in early biofilm supernatants were detected and annotated in untargeted NMR data analysis, followed by *in silico* examination of the biochemical relationships between the metabolites and cellular processes. Studies on metabolic exchanges among commensal dental biofilm bacteria and interactions with host components such as salivary glycoproteins are of importance for better understanding the formation and maturation of oral microbial ecosystems and their roles in oral health and disease. The early establishment of commensals is an important mechanism of the innate immune defense to stabilize the microbial ecosystem of the host and prevent the colonization of external pathogens.

In monospecies early biofilms, *Streptococcus gordonii* adhered significantly better to salivary MUC5B compared to *Actinomyces naeslundii*. In dual species, the surface coverage and biomass of attached cells were similar to the *S. gordonii* monospecies biofilms and higher than the *A. naeslundii* biofilms, with an equal distribution of the two species. This suggests a co-adhesion relationship where the colonization of *A. naeslundii* on salivary MUC5B is promoted by the presence of *S. gordonii* during early biofilm attachment. Numerous previous studies have found that co-aggregation between *Actinomyces* and *Streptococcus* occurs through specific cell surface protein and/or polysaccharide interactions (18, 21, 33 – 35), and that the spatial proximity that is established during these interactions enables mutually favorable interspecies cooperation in, e.g., metabolism of nutrients, biofilm formation and growth, and modification of local microenvironments, all of which may increase the competitiveness of these species in mixed biofilms (21). The biofilm model used in this study allowed for investigation of bacterial interactions and adaptations during early biofilm formation in the presence of salivary MUC5B, in an effort to increase the similarity to *in vivo* conditions compared to traditional planktonic models in growth medium without components from human saliva.

The complementary glycosidase profiles displayed by the two commensals showed potential for synergistic degradation of MUC5B carbohydrate chains, which would make the complex salivary MUC5B more readily available as a nutrient source during biofilm establishment and growth. Previous studies have shown that *A. naeslundii* possesses some carbohydrate uptake and glycolytic enzymes that differ from those of *Streptococcus*, namely the phosphorylating enzymes GTP/polyphosphate (PPn)-dependent glucokinase, pyrophosphate (PPi)-dependent phosphofructokinase, UDP-glucose pyrophosphorylase, and GDP/IDP-dependent PEP carboxykinase (20). The utilization of non-ATP (PPi) phosphoryl donors in carbon uptake and metabolism as well as secondary conversion of lactate to acetate and more efficient intracellular polysaccharide production by use of the highly active intracellular UDP-glucosepyrophosphorylase (compared to *Streptococcus* which uses ADP-glucose pyrophosphorylase for intracellular polysaccharide (IPS) synthesis) are thought to contribute to the efficient and flexible carbon metabolism in *A. naeslundii* and increase its competitiveness in mixed oral biofilms (19, 20). The majority of the metabolites that were found in the NMR analysis (acetate, acetone, butyrate, ethanol, formate, lactate, methanol, propionate, pyruvate, and succinate) are well-known products of carbohydrate metabolism in oral bacteria, indicating that MUC5B elicited a metabolic response in the bacteria and that the bacteria were able to degrade and utilize monosaccharide moieties from the mucin oligosaccharide chains as a carbon source during early biofilm formation. The identified differences between the monospecies and dual species metabolomes showed that the two species were able to take advantage of each other’s enzymatic complementarity and perform secondary metabolic interactions. Such interspecies cooperation in the utilization of complex glycoproteins as nutrients has been found to promote health-associated diversity in resident oral biofilms (36, 37).

Since the metabolome in these experiments was studied in early biofilm supernatants, for preservation of cellular energy, the amino acids and amino acid (leucine) degradation product that were identified (valine, glutamate, and arginine and 2-oxoisocaproate) are more likely to originate from extracellular degradation of the MUC5B protein core, rather than from exocytosis after intracellular amino acid biosynthesis. The mucin subunits of salivary MUC5B are highly glycosylated, but also contain C- and N-terminal, non-glycosylated regions of exposed polypeptide backbone that may be available for utilization as nutrients for bacteria with proteolytic capacity for amino acid release and uptake from this substrate (15). These regions are rich in a variety of amino acids such as proline, serine, and threonine, but also contain the amino acids and degradation product precursor identified in the monospecies and dual species early biofilm metabolomes (13) (UniProt accession number Q9HC84). Based on the current findings, *A. naeslundii* and *S. gordonii* clinical isolates were able to release some amino acids from the mucin protein core. The differences between monospecies and dual species metabolomes that were identified also indicated that the two species were able to cooperate not only to hydrolyze and metabolize polysaccharide moieties, but also to release MUC5B polypeptide amino acids.

Acetate concentrations in the dual species biofilms were higher than what would be expected from the sum of the two monospecies biofilms, while lactate concentration was lower. In oral saccharolytic bacteria such as *Actinomyces* and streptococci, acetate and lactate are both products of pyruvate conversion (20, 38). In oral streptococci, pyruvate, largely produced from glycolytic (Embden-Meyerhof-Parnas pathway) degradation of carbohydrate substrates, is under circumstances of limited environmental sugar concentrations, such as in the present study, converted through heterofermentation to various weak organic acids such as acetate and formate, as well as ethanol and low amounts of lactate, which are then released from the cells as metabolic end products (38). Under conditions of sugar excess in the oxygen-limited oral biofilms, such as after host intake of carbohydrate-rich food, streptococci can switch to homolactic fermentation by switching from using pyruvate formate lyase to lactate dehydrogenase for pyruvate conversion, in order to speed up the capacity to flux carbohydrates through glycolysis for efficient regeneration of NAD and prevention of accumulation of cytotoxic intermediates which may otherwise cause the cell to lyse (32, 38, 39). This causes increased accumulation of the more acidic lactic acid which rapidly lowers the local pH, allowing streptococci to outcompete other species that are acid sensitive and over time contribute to the development of dental caries lesions (38). Unlike streptococci, *Actinomyces* do not show an apparent shift of fermentation pattern in response to environmental excess or limited glucose conditions (20, 40), which might explain the increased accumulation lactate in monospecies biofilms of this species compared to *S. gordonii*. However, in mixed biofilms, oral species of this genera have been found to metabolize lactate into acetate (20), and thereby help reduce the acidity of oral biofilms. The results of this study indicate that when *A. naeslundii* and *S. gordonii* form biofilms together in the presence salivary MUC5B, synergistic regulation of pyruvate conversion pathways in one or both species, and/or secondary metabolism of lactate to acetate by *A. naeslundii*, occurs. As a consequence, it can be hypothesized that MUC5B supports the establishment of more diverse and less cariogenic dental biofilms through enabling co-adherence as well as providing a nutrient source for synergistic utilization by the oral commensals *S. gordonii* and *A. naeslundii* on MUC5B. Thereby, salivary MUC5B may act as a part of innate immune mechanisms to regulate oral biofilm activity toward maintaining biosis.

The metabolites that were produced by all three groups but with no significant changes in abundance in the dual species early biofilms (formate, ethanol, and unidentified metabolites 11, 23, 69, 38, 71, 72, 40) represent a shared core metabolic output profile that is seemingly unaffected by dual species culturing. The mapping of core metabolomes shared across species are of interest for future characterization of metabolic patterns that may deviate from this pattern. Identification of distinct metabolomic patterns that reflect microbial activities will facilitate further understanding of the establishment and maturation of commensal microbial ecosystems in the host.

Butyrate, succinate, and 2-oxoisocaproate were exclusively produced by *A. naeslundii*, and arginine*, methanol, and propionate by *S. gordonii*. These metabolites were also found in the dual species biofilms with abundances unaffected by the presence of the other species, except for succinate which was more abundant in the *A. naeslundii* monospecies biofilms compared to in the dual species, suggesting that some synergistic effect seems to cause a reduced accumulation of this metabolite in the dual species biofilms. Some gut bacteria that are also present in the mouth such as *Veillonella* spp. can convert succinate to propionate through the succinate-propionate pathway (41). It is possible that succinate was co-metabolized further to propionate by *S. gordonii* in the dual species biofilm; however, the enzymes for this pathway are not well studied and have not been annotated in *S. gordonii* (42). It is also possible that when *A. naeslundii* and *S. gordonii* attach together, *S. gordonii* uses up the nearby carbon dioxide more efficiently and thereby reduces the available carbon sources needed for *A. naeslundii* succinate production. Succinate from oral biofilms has been found to disturb the host immune system in gingival epithelium (43) and may promote the establishment of periodontal pathogens such as *Porphyromonas gingivalis* (44). Secondary metabolism of succinate may thereby contribute to preventing transition to periodontopathogenic dysbiosis in oral biofilms.

The main metabolites that differentiated *A. naeslundii* monospecies biofilms from *S. gordonii* and dual species biofilms in the PCA-based covariation analysis were 2-oxoisocaproate, 57, succinate, 38, acetate, butyrate, 56, 55, lactate, 45, and pyruvate. Five of these (succinate, acetate, butyrate, lactate, and pyruvate) are involved in pyruvate metabolism (carbon metabolism). *S. gordonii* biofilms were mostly differentiated by propionate, formate, ethanol, 39, 71, glutamate, methanol, and arginine*. Three of these (propionate, formate, ethanol) are involved in pyruvate metabolism and two (glutamate, arginine*) are amino acids. As expected, the overall dual species biofilm metabolome was situated between those of the individual monospecies early biofilms on the PCA; however, there were some specific metabolite relationships that differentiated the dual species biofilms from the two monospecies. Valine was found only in the dual species early biofilms while missing from both monospecies biofilms, and acetone and metabolite 70 were missing from the dual species biofilm while present in both monospecies biofilms. Pyruvate and metabolites 45, 55, 56, 57 were present only in the *A. naeslundii* monospecies biofilms, and glutamate and metabolite 39 were present only in *S. gordonii*, and missing from the dual species biofilms. This indicates that there were synergistic effects of either metabolite co-regulation or secondary metabolic interplay in metabolic pathways between the two species that include these metabolites. The pathway that was found to be significantly over-represented in the pathway enrichment analysis of all metabolites identified in *A. naeslundii, S. gordonii,* and dual species early biofilm formation in the presence of MUC5B was pyruvate metabolism. This is not surprising, since this pathway is central in the metabolism of both carbohydrates and amino acids, and constitutes a junction point between these metabolic chains.

The study of host mechanisms that maintain oral health through salivary-mediated regulation of oral biofilm formation and activity is of importance for understanding oral biofilm maturation. Effects on metabolomic profiles in oral commensals in response to salivary MUC5B may be associated with regulatory events for sustaining homeostasis in oral biofilms. Further studies are needed to clarify these relationships, and studies on the roles of salivary MUC5B on biofilm maturation, succession, diversity, and growth in the longer time perspective are of great interest. Experiments with addition of spent media from biofilms grown with salivary MUC5B to other species over time could be helpful to elucidate the specific effects of metabolites from degradation of this complex glycoprotein on biofilm maturation and composition. Further developments in gene sequencing, annotation, and expression of oral species are needed to allow for more detailed analyses such as glycosidase profiling with technologies such as qPCR or RNA-Seq. Such studies would greatly contribute to increasing the understanding of complementarity of different oral species during degradation of complex substrates. New findings in these areas may contribute to the development of improved prevention strategies against biofilm-induced disease in the future.

### Conclusions

The two early colonizing oral commensals *A. naeslundii* and *S. gordonii* demonstrated a co-adhesion relationship during early biofilm formation in the presence of salivary MUC5B, where the colonization of *A. naeslundii* on salivary MUC5B was facilitated by *S. gordonii*. Moreover, *A. naeslundii* and *S. gordonii* were able to utilize salivary MUC5B as a sole nutrient source during early biofilm formation. The metabolic end products that constituted the metabolome suggested that the bacteria were able to release mucin carbohydrates from oligosaccharide sidechains as well as amino acids from the protein core. Finally, synergistic effects were seen in the dual species biofilm metabolome compared to the monospecies, showing that *A. naeslundii* and *S. gordonii* cooperated in degrading salivary MUC5B. A better understanding of bacterial interactions and salivary-mediated regulation of early dental biofilm activity is meaningful for understanding oral biofilm physiology and may contribute to the development of prevention strategies against biofilm-induced disease in the future.

## Data Availability

The metabolomics data has been deposited in the MetaboLights online repository (45) with the data set identifier MTBLS8370.
